# Cervical Agenesis and Compressive Hydronephrosis in a Patient With Limited English Proficiency

**DOI:** 10.7759/cureus.114525

**Published:** 2026-08-14

**Authors:** Charolette Wollermann, Alysse Alejandro-Hernandez, Shannon Schellhammer, Steve Carlan

**Affiliations:** 1 Obstetrics and Gynecology, Orlando Regional Medical Center, Orlando, USA; 2 Obstetrics and Gynecology, Orlando Regional Medical, Orlando, USA

**Keywords:** acute pelvic pain, cervical agenesis, endometriosis, hydronephrosis, primary amenorrhea, retrograde menstruation

## Abstract

Cervical agenesis is an extremely rare congenital anomaly of the female reproductive tract characterized by normal fallopian tubes, an absent cervix, a functioning uterine body, and, occasionally, partial or total vaginal aplasia. It often presents with primary amenorrhea and cyclic pelvic pain. Delayed diagnosis can lead to severe morbidity from retrograde menstruation, including hematometra, hematosalpinx, and endometriosis. Patients with limited English proficiency (LEP), particularly Haitian women, face compounded systemic hurdles when navigating the healthcare system, significantly increasing the risk of nonattendance at follow-up appointments. A 27-year-old Haitian Creole-speaking nulligravida woman with LEP presented with a multi-year history of worsening cyclic pelvic pain and primary amenorrhea. Over 951 days, she sought care 16 times across multiple EDs, imaging centers, and healthcare clinics. Despite the consistent presence of a medical interpreter and repeated cross-referrals, the patient successfully attended only three general gynecology clinic visits and did not complete the necessary reproductive endocrinology subspecialty appointments, highlighting a breakdown in both follow-up and systemic tracking mechanisms. Diagnostic imaging eventually revealed severe hematometra, bilateral hematosalpinx, and large pelvic endometriomas. The untreated, chronic retrograde menstruation led to mass-effect compression of the left ureter, causing rapidly progressive unilateral hydronephrosis. Without treatment, cervical agenesis with a functioning endometrium can cause profound anatomical distortion and secondary urological complications. This case underscores the danger of relying on passive referral systems for vulnerable LEP patient populations. LEP is one of several contributing factors in delayed care and highlights the critical need for active, language-concordant patient navigation to prevent catastrophic breakdowns in continuity of care. Patient participation in the referral process is critical.

## Introduction

Congenital obstructive genital tract anomalies are rare but clinically significant causes of amenorrhea, pelvic pain, and infertility [1]. Cervicovaginal agenesis with functioning endometrium is one of the least common congenital anatomic reproductive anomalies, with a reported worldwide incidence of 1/80,000 to 1/100,000 [2]. This condition results in primary amenorrhea and ultimately hematometra, hematosalpinx, and endometriosis [2]. Cervical agenesis is typically associated with a functioning uterus and may occur with normal fallopian tubes and, in 50% of cases, partial or total vaginal aplasia [3].

A medical workup is indicated at the time of confirmation of primary amenorrhea, defined as the absence of menstruation by age 15 years or three years after the onset of breast development (thelarche) [4]. The diagnosis of cervicovaginal agenesis requires a thorough history and physical examination, pregnancy testing, serum testosterone and follicle-stimulating hormone levels, a karyotype, and pelvic ultrasound, magnetic resonance imaging (MRI), and, occasionally, computed tomography (CT). The differential diagnosis includes vaginal obstruction due to transverse vaginal septum, imperforate hymen, and distal vaginal atresia. It also includes obstructions associated with Müllerian fusion defects and differences in sexual development, including gonadal dysgenesis, complete androgen insensitivity, and congenital adrenal hyperplasia due to 17α-hydroxylase deficiency [5]. The diagnostic process often involves coordination among an interprofessional team of providers, but ultimately a reproductive endocrinologist may lead the treatment team.

Clinical execution is frequently derailed when patients face socio-linguistic barriers. Immigrants with limited English proficiency (LEP) face multiple significant hurdles when interfacing with the United States (US) healthcare system. These challenges can lead to increased costs, anxiety, and delayed care [6]. Miscommunication, impaired patient-provider trust, unfamiliar cultural paradigms, and fragmented care coordination across separate health systems pose formidable challenges to achieving definitive clinical resolution, especially for chronic and complex conditions [6-8]. These barriers are uniquely pronounced among Haitian immigrants, who experience profound linguistic isolation and interpretation deficits within standard medical networks [6].

We present a case of an adult Haitian woman with LEP and cervical agenesis whose definitive care was delayed for nearly three years across multiple health systems. The delay culminated in compressive unilateral hydronephrosis.

## Case presentation

A 27-year-old Haitian Creole-speaking nulligravida woman presented to our gynecology clinic on Day 0 with severe intermittent pelvic pain and deep-thrust dyspareunia that had progressed over several months. Her past medical history was notable for normal secondary sexual characteristics, with thelarche and pubarche occurring at ages 11-12 years, but for the complete absence of menarche. She was a temporary protected status holder with unrestricted work authorization, was married and in a stable relationship, held full-time employment at a local resort, and had private medical insurance. She denied tobacco, alcohol, or illicit substance use, had no prior surgical history, took no regular medications, and had no known family history of congenital anomalies or gynecological malignancies.

**Figure 1:**
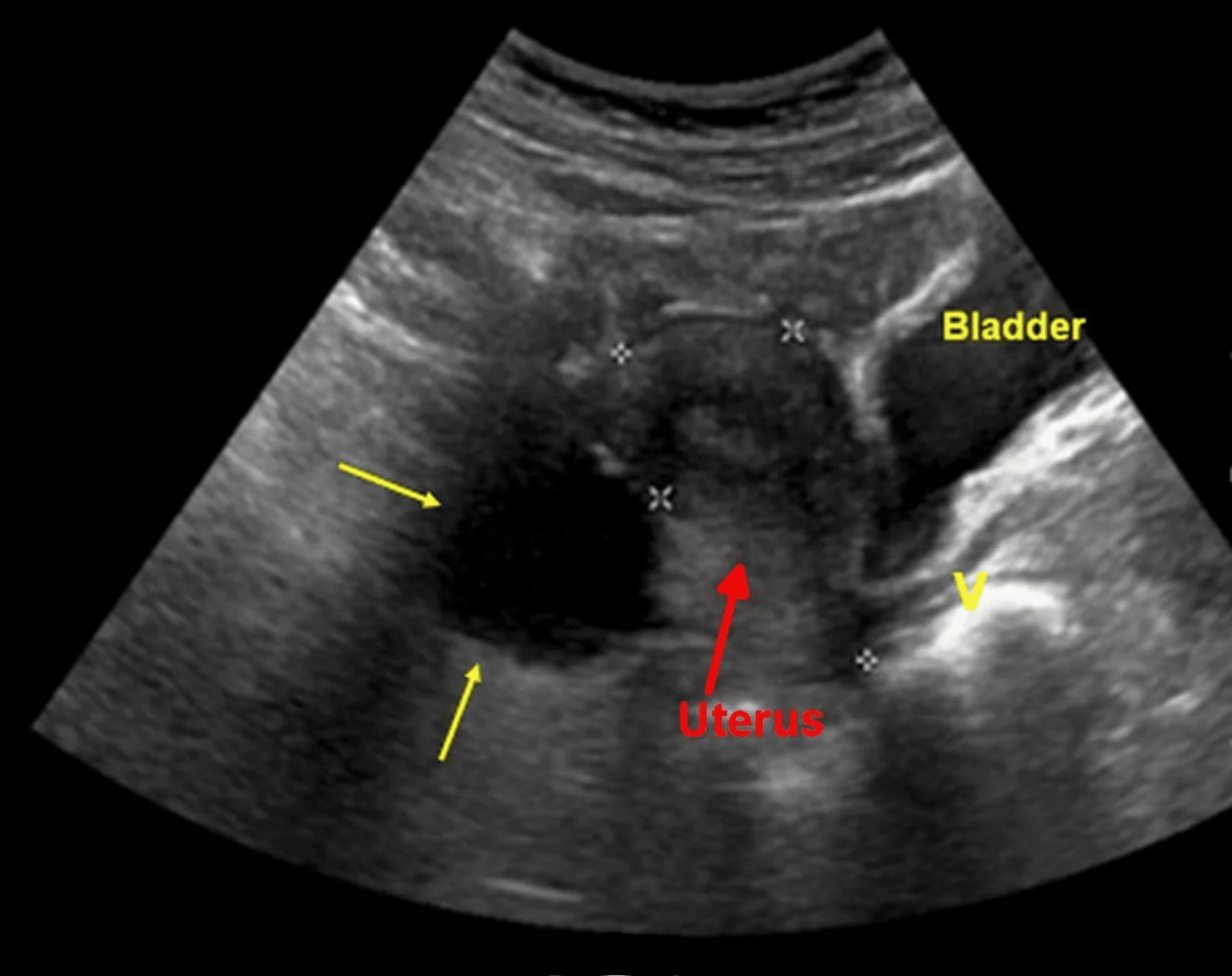
Initial transabdominal pelvic ultrasound The uterus, indicated by the cursors, measures 5.59 × 3.0 cm (red arrow). The yellow arrows indicate a clear cystic structure posterior to the uterus, with associated acoustic shadowing. No free fluid or evidence of pregnancy is identified.

Approximately 22 months before Day 0, she sought care at a primary family practice clinic for ongoing pelvic pain, and a transabdominal ultrasound was performed (Figure 1).

Routine screening and baseline laboratory tests, including a complete blood count (CBC) and a comprehensive metabolic panel (CMP), were ordered but not completed due to patient-reported transportation barriers (Tables 1-2).

**Table 1:** Summary of clinical encounters, diagnostic testing, and examination findings CBC: complete blood count, CMP: comprehensive metabolic panel, CT: computed tomography, ED: emergency department, HCG: human chorionic gonadotropin, MRI: magnetic resonance imaging, STDs: sexually transmitted diseases, TVUS: transvaginal ultrasound, UA: urinalysis

Relative time	Age	Test	Translator	Chief complaint/purpose of visit	Physical findings
−26.4 months	25	TVUS	Cousin	Pelvic pain, primary amenorrhea	No examination documented
−22.4 months	25	Given orders for: CBC, CMP, STD screening	Not documented	Pelvic and abdominal pain	No examination documented
−20.0 months	25	Annual examination	Family member	To review labs and annual exam	No abnormalities documented
−19.4 months	25	MRI, pelvis	Not documented	To review MRI	Telemedicine consult
−15.7 months (admitted 2 days and seen by gynecology while an inpatient)	26	CT, abdomen/pelvis with contrast, TVUS	Translation services	Lower abdominal pain	Pelvic examination "deferred" per records
−15.4 months	26	Office consultation	Translation services	Post hospital follow-up visit	No pelvic exam documented
−13.4 months	26	Care from outside healthcare system	Not documented	Not documented	Not documented
−3.9 months	27	Care from outside healthcare system	Translation services	Follow-up to review MRI	No pelvic exam documented
−3.1 months	27	TVUS	Translation services	Pelvic pain	Cervix not visualized, uterus tender, adnexal masses
−3.0 months	27	Gynecology office consultation with examination	Translation services	Follow-up visit from hospital discharge	No cervix palpable or visualized on speculum exam
−2.2 months	27	Labs and TVUS	Family member	Abdominal pain	No pelvic exam documented
−2.0 months	27	Gynecology office consultation without examination	Translation services	Follow-up from ED visit	No pelvic exam documented
−11 days	27	MRI, pelvis	Not documented	Referral for imaging	No pelvic exam documented
Day 0	27	Gynecology office consultation	Translation services	Pelvic pain and infertility	No pelvic exam documented
+3 months	27	Negative urine and serum pregnancy tests	Hospital interpreter	Pelvic pain	Pelvic exam by nurse confirmed previous findings
+5.5 months	27	CBC, CMP, HCG, urinalysis (all negative)	Hospital interpreter	Pelvic pain	No pelvic exam documented

**Table 2:** Summary of imaging results, clinical diagnoses, and recommended follow-up CT: computed tomography, MRI: magnetic resonance imaging, OB/GYN: obstetrics and gynecology, TVUS: transvaginal ultrasound, ED: emergency department, RLQ: right lower quadrant

Relative time	Imaging	Findings	Location of point of care	Discharge diagnosis	Post-visit instructions
−26.4 months	TVUS	Not available	ED	Ovarian cyst and thickened endometrium	Follow-up with gynecologist; she did not keep the appointment
−22.4 months	No	All negative	Family medicine clinic	Primary amenorrhea	Follow-up with gynecologist; she did not keep the appointment
−20.0 months	No	Negative pregnancy test	Family medicine clinic	Primary amenorrhea	Follow-up with gynecologist; she did not keep the appointment
−19.4 months	MRI, pelvis	Enlarged bilateral ovaries, likely endometriomas	Telemedicine with family medicine	Endometrioma	Follow-up with gynecologist; she did not keep the appointment
−15.7 months (admitted 2 days and seen by gynecology while an inpatient)	CT, abdomen/pelvis with contrast, TVUS	Cystic and solid bilateral adnexal multiloculated lesions. Normal serum prolactin, testosterone, gonadotropins	ED and hospital	RLQ pain, resolved. Bilateral ovarian cysts	Follow-up with gynecologist and reproductive endocrinology; she did not keep the appointment
−15.4 months	No	Patient is asymptomatic at this time. Vitals are stable	STAR clinic internal medicine	Bilateral cysts and primary amenorrhea	Follow-up with gynecologist; she did not keep the appointment
−13.4 months	TVUS	Large amount of debris within the endometrium. Right ovary with differential including endometrioma. Multi-septated cystic structure in the left ovary	AdventHealth	Probable endometriomas, bilateral ovaries	The above findings should be further evaluated with a gynecologic evaluation and pelvic MRI
−3.9 months	MRI, pelvis	Bilateral ovarian cysts. Cervix is not well visualized	Women's healing clinic	Cervical agenesis and ovarian cysts	Follow-up with gynecologist; she did not keep the appointment
−3.1 months	TVUS	Bilateral ovarian masses, endometriomas. Negative urine pregnancy test	ED and hospital	Ovarian cyst versus mass versus endometrioma versus pregnancy	Referred to reproductive endocrinology, did not follow up. Referred to gynecology. Made appointment.
−3.0 months	No	-	Kept appointment at the outpatient gynecology clinic	Primary amenorrhea	Counseled patient regarding exam findings; patient voiced understanding
−2.2 months	TVUS	No change in bilateral ovarian complex cysts	ED and hospital	Endometriosis	Referred to OB/GYN
−2.0 months	No	Counseled to follow up with requested pelvic MRI	Kept appointment at the outpatient gynecology clinic	Primary amenorrhea, infertility, and suspected congenital anomaly of reproductive tract	MRI and reproductive endocrinology referral
−11 days	MRI pelvis with and without contrast	Bilateral hematosalpinx and hematometra. Findings are suggestive of cervical hypoplasia versus agenesis	Imaging center	Findings are suggestive of cervical hypoplasia versus agenesis	Follow-up with gynecology clinic
Day 0	No	Unchanged	Kept appointment at the outpatient gynecology clinic	Primary amenorrhea, pelvic pain, hematometra, hematosalpinx	Discussed results of MRI. Follow-up with reproductive endocrinology
+3 months	CT abdomen pelvis with contrast, TVUS	Severe bilateral hematosalpinx, left moderate hydroureteronephrosis	ED	Hematosalpinx and left hydronephrosis without urolithiasis	Discussed with her to return to ED with any new or worsening symptoms
+5.5 months	No	Negative serum pregnancy test	ED	Unchanged	Given instructions to follow up with gynecologist

Approximately 20 months before Day 0, she returned to the primary clinic and was prescribed naproxen 500 mg daily for cyclic pelvic pain. A pelvic MRI performed approximately 19 months before Day 0 demonstrated enlarged bilateral ovaries with complex cysts measuring up to 7.6 cm on the right, highly suggestive of endometriomas, a left hydrosalpinx, and a markedly distended endometrial canal containing hyperintense material consistent with retained blood products.

**Figure 2:**
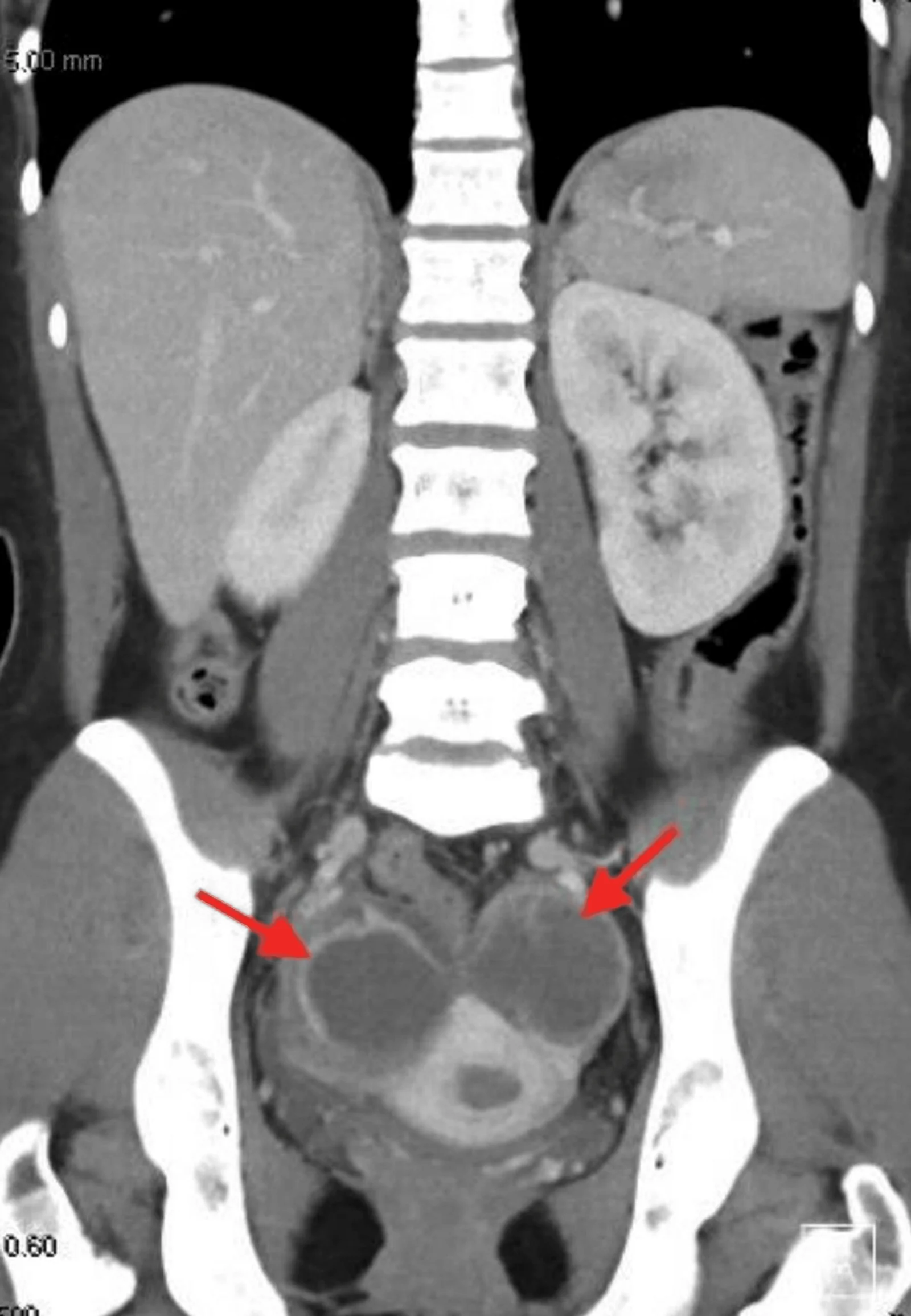
Coronal contrast-enhanced CT showing bilateral adnexal lesions Cystic and solid multiloculated bilateral adnexal lesions (red arrows), measuring up to 7.6 × 7.8 cm on the right. Correlation with pelvic ultrasound is recommended. CT: computed tomography

**Figure 3:**
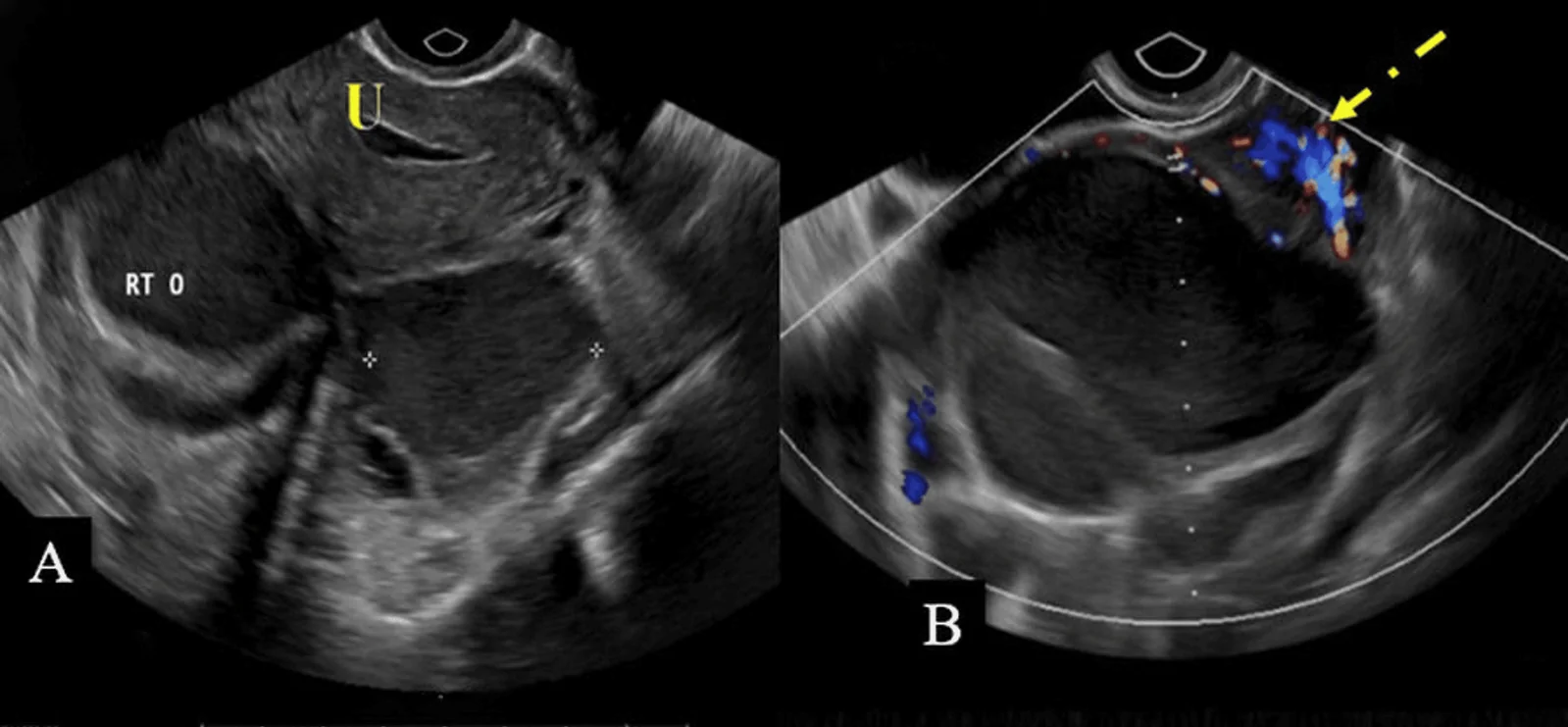
TVUS demonstrating bilateral ovarian abnormalities "A" shows both ovaries on a transverse scan, "Rt O" shows the right ovary, "U" shows the uterus, and "B" shows color Doppler imaging with vascular flow in the capsule (hatched yellow line) and no vascular flow within the ovary itself. The left ovary measures 3.74 cm. Low-level echoes within the ovarian tissue are consistent with hemorrhagic cysts or endometriomas. TVUS: transvaginal ultrasound

Approximately 15 months before Day 0, she was admitted to the hospital through the ED for intractable pelvic pain. A repeat CT scan (Figure 2) and transvaginal ultrasound (TVUS) (Figure 3) corroborated the stable, large pelvic masses and retained endometrial fluid. Endocrine serum evaluations revealed a normal hormone profile (Table 3).

**Table 3:** Serum hormonal evaluation

Test (serum)	Reference range	Patient value
Free testosterone	0.13-1.06 ng/dL	0.32 ng/dL
Total testosterone	8-60 ng/dL	9.7 ng/dL
Beta-human chorionic gonadotropin	Negative	Negative
Follicle-stimulating hormone	1.4 to 9.9 mIU/mL (follicular phase)	3.8 mIU/mL
Luteinizing hormone (serum)	1.9 to 14.6 mIU/mL (follicular phase)	3.44 mIU/mL
Prolactin	2-30 ng/mL	32 ng/mL

Although appointments with reproductive endocrinology and infertility (REI) and gynecology were generated upon discharge, she did not attend either. A post-hospitalization check-up at the primary care clinic approximately 15 months before Day 0 noted that she was temporarily asymptomatic, and a repeat referral to gynecology was made but not kept. Following a subsequent TVUS at an outside facility approximately 13 months before Day 0, the patient was lost to follow-up for approximately nine months.

Approximately four months before Day 0, she attended a consultation at an outside community health clinic, where an external MRI report noted for the first time that the uterine cervix was “not well visualized.” Approximately three months before Day 0, she returned to our ED with acute pain. For the first time during her two-year clinical course, a physical pelvic examination was documented in the medical records, revealing a completely blind-pouch vaginal vault with an absent cervix and a tender, palpable uterine corpus.

**Figure 4:**
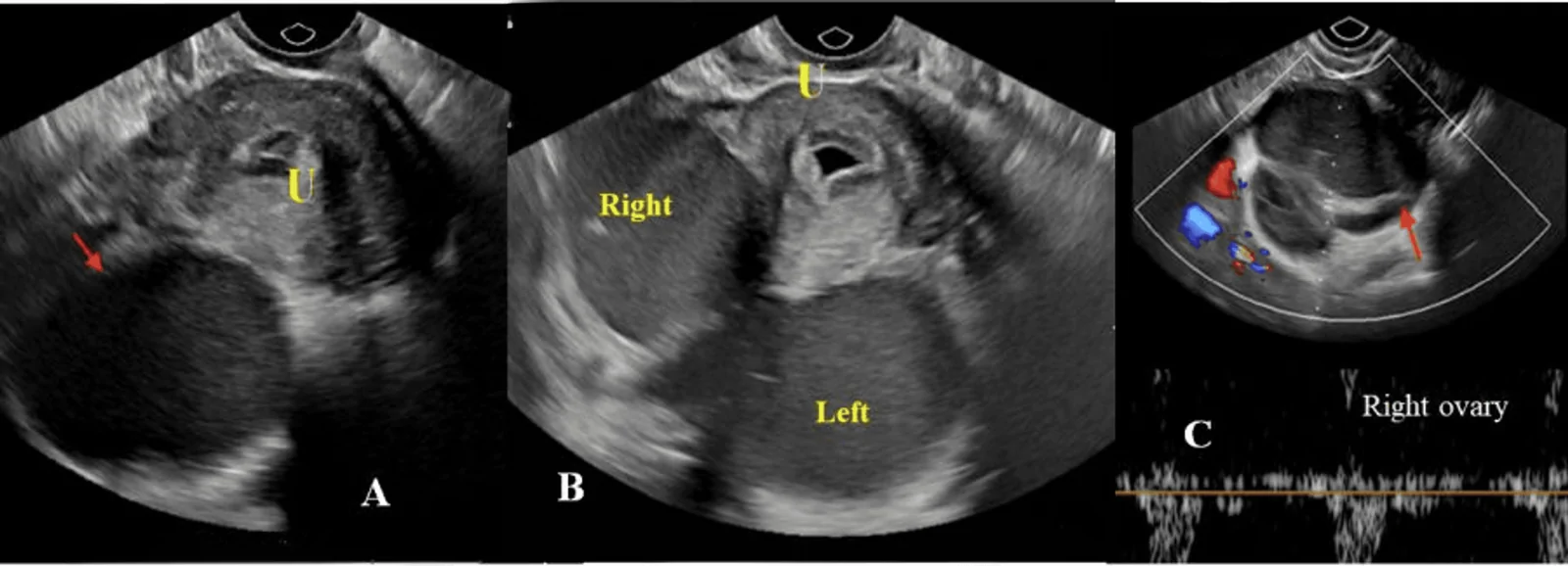
TVUS demonstrating bilateral complex ovarian lesions "A" shows a longitudinal scan showing the uterus ("U") and an ovarian cyst (red arrow) in the cul-de-sac, and "B" shows a transverse scan showing the right ovary superior to the left ovary. The right ovary measures 7.6 × 6.0 × 5.6 cm, and the left ovary measures 5.2 × 3.7 × 2.4 cm. Both ovaries demonstrate homogeneous echogenicity with low-level echoes, interpreted as either hemorrhagic cysts or endometriomas. "C" shows a color Doppler image of the right ovary showing venous flow within the capsule and no vascular flow within the ovary itself. The red and blue colors represent blood flow within the vascular structures, and the red arrow indicates one of the cystic cavities. TVUS: transvaginal ultrasound

A concurrent TVUS confirmed no changes from the previous scan, with persistent complex pelvic fluid collections (Figure 4). She attended a follow-up appointment at our gynecology clinic three days later, where a formal physical examination confirmed normal secondary sexual development but an absent cervix, without a vaginal septum or visible vault anomalies. A repeat high-priority referral to REI was made. However, approximately two months before Day 0, she returned to the ED for acute abdominal pain.

**Figure 5:**
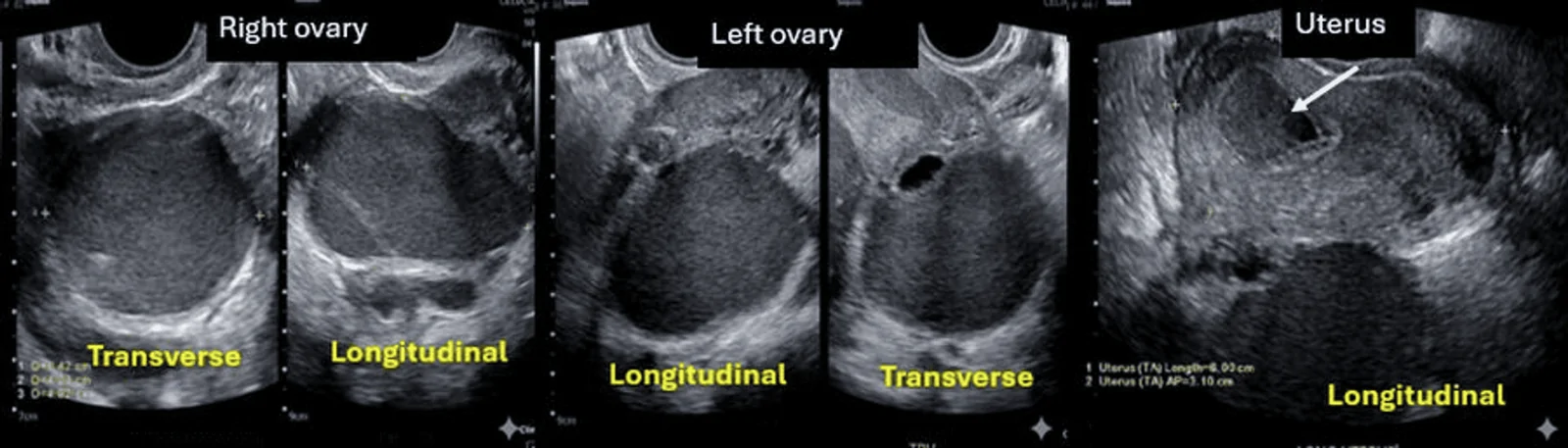
Follow-up TVUS of the adnexa and endometrial cavity Transverse and longitudinal views of the right and left adnexa demonstrate no interval change in the bilateral complex ovarian cysts, which most likely represent endometriomas. Less benign cystic lesions cannot be excluded. A probable dilated fallopian tube adjacent to the right complex cyst may also contain blood products. No hyperemia is observed to suggest an abscess, and there is no evidence of ovarian torsion. The uterus is visualized. Complex fluid distends the fundal endometrial cavity (white arrow), possibly representing blood products. TVUS: transvaginal ultrasound

**Figure 6:**
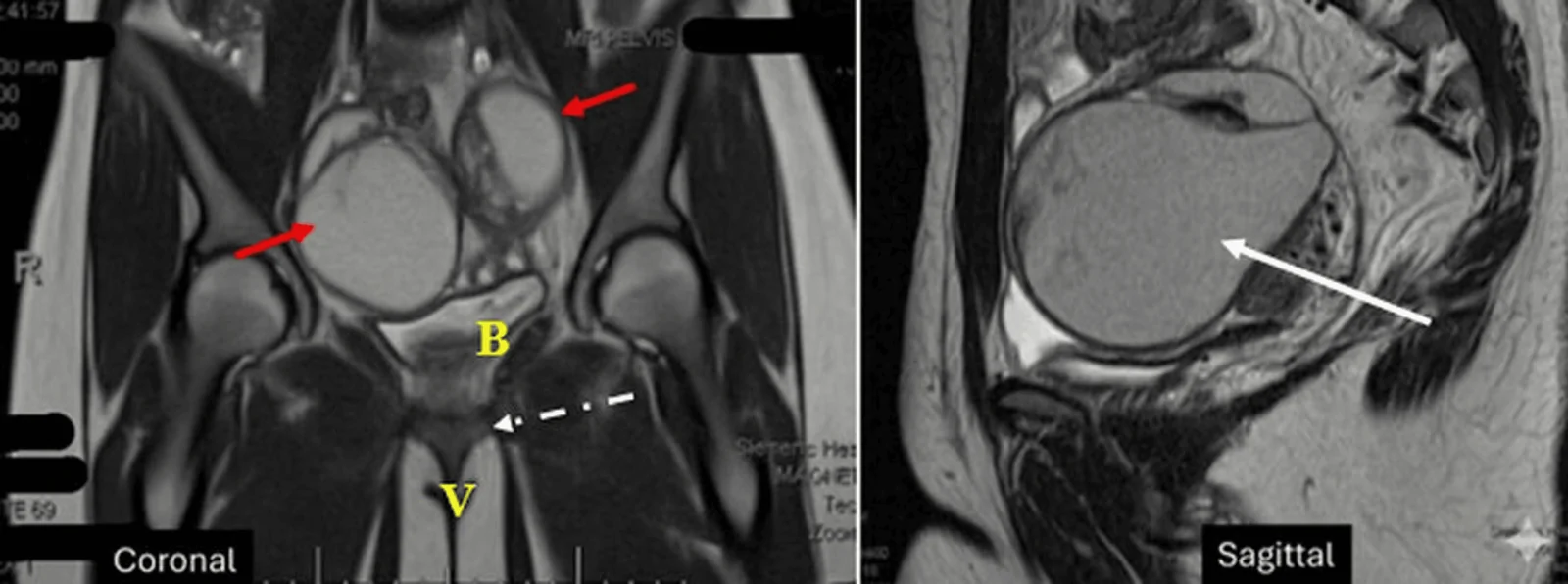
T2-weighted pelvic MRI demonstrating suspected outflow obstruction Coronal scan demonstrating possible bilateral hematosalpinx (red arrows) versus endometriomas. "B" indicates the bladder. The findings are suggestive of cervical hypoplasia versus agenesis; however, this image alone is insufficient to diagnose cervical agenesis. The absence of a clearly visualized cervical structure in this coronal slice may be attributable to severe anatomic distortion and the limitations of a single two-dimensional plane. Given the abrupt caliber change at the level of the possible internal os and the upper vagina (dotted white line), associated septa or webs cannot be completely excluded, although manual examination revealed no vaginal anatomic abnormality. The sagittal scan demonstrates possible hematometra (white arrow). MRI: magnetic resonance imaging

A repeat TVUS demonstrated unchanged, persistent findings highly suggestive of large bilateral endometriomas or a severely dilated hemorrhagic fallopian tube adjacent to a complex ovarian cyst (Figure 5). Five days later, she returned to the gynecology clinic for follow-up planning, where referrals for a repeat confirmatory pelvic MRI and another REI consultation were generated. The confirmatory pelvic MRI was performed approximately 11 days before Day 0, explicitly revealing large bilateral masses accompanied by hematometra, with structural findings highly suggestive of congenital cervical agenesis versus severe hypoplasia (Figure 6).

At the Day 0 gynecology visit, the patient complained of infertility and deep-thrust dyspareunia without bleeding. A bimanual examination revealed a large, tender, palpable mass behind the posterior vaginal wall, centered in the left adnexa. Because the patient was hemodynamically stable, afebrile, and voiding without difficulty, she was placed on continuous oral contraceptive pills to suppress menstruation and alleviate retrograde flow. Another high-priority referral to REI was submitted, and a three-month gynecologic follow-up was scheduled. The patient did not attend the REI appointment. Approximately three months after Day 0, she returned to the ED with agonizing pelvic pain.

**Figure 7:**
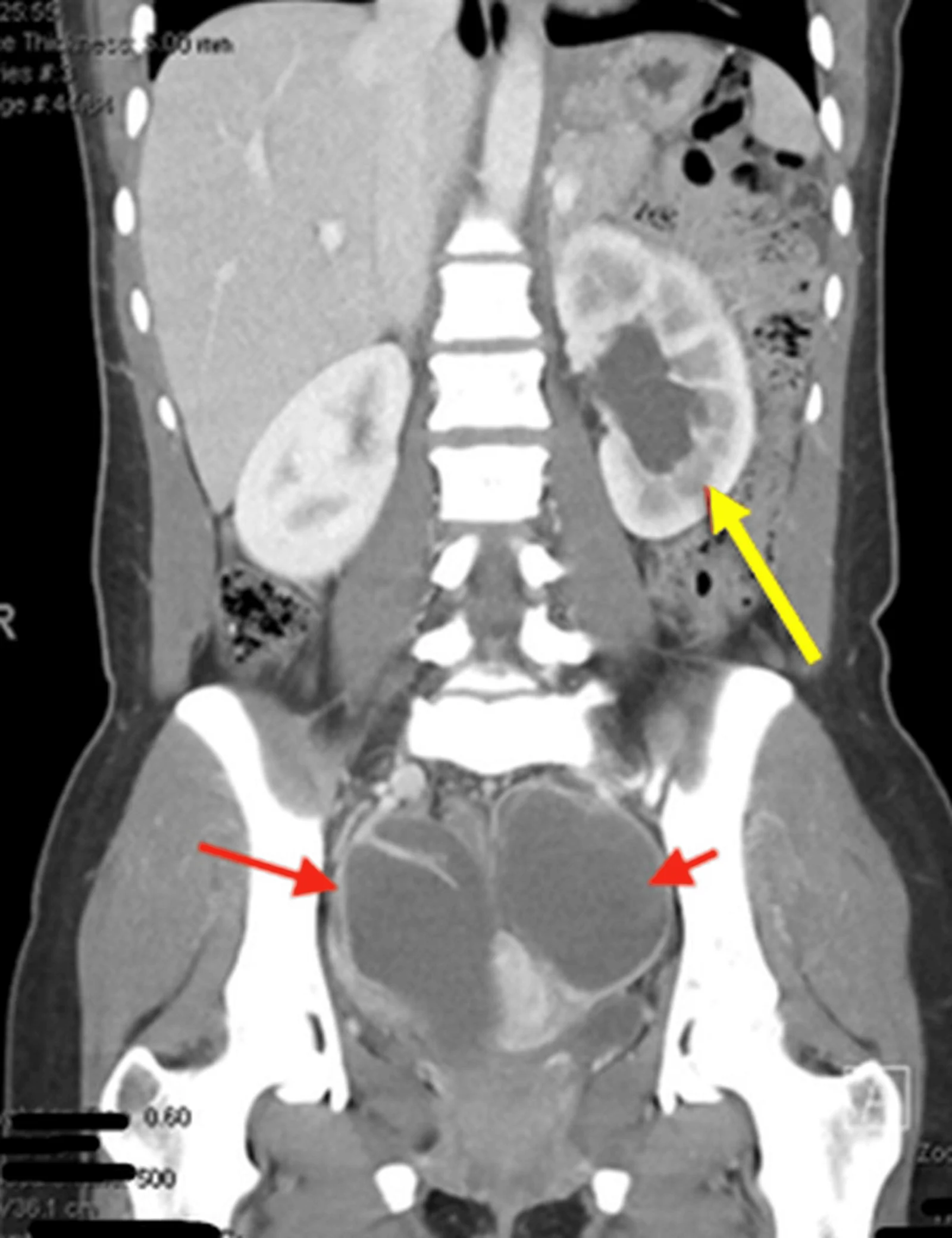
Contrast-enhanced CT demonstrating left hydronephrosis and pelvic mass effect Renal changes presumed to be secondary to severe bilateral hematosalpinx. The findings suggest that the obstruction is extrinsic rather than due to an internal urinary calculus. The lower red arrows indicate two large, distinct fluid-filled pelvic masses that appear to compress the ureters externally, resulting in impaired urinary drainage. These masses are favored to represent hematosalpinx rather than ovarian pathology, including endometriomas. CT: computed tomography

**Figure 8:**
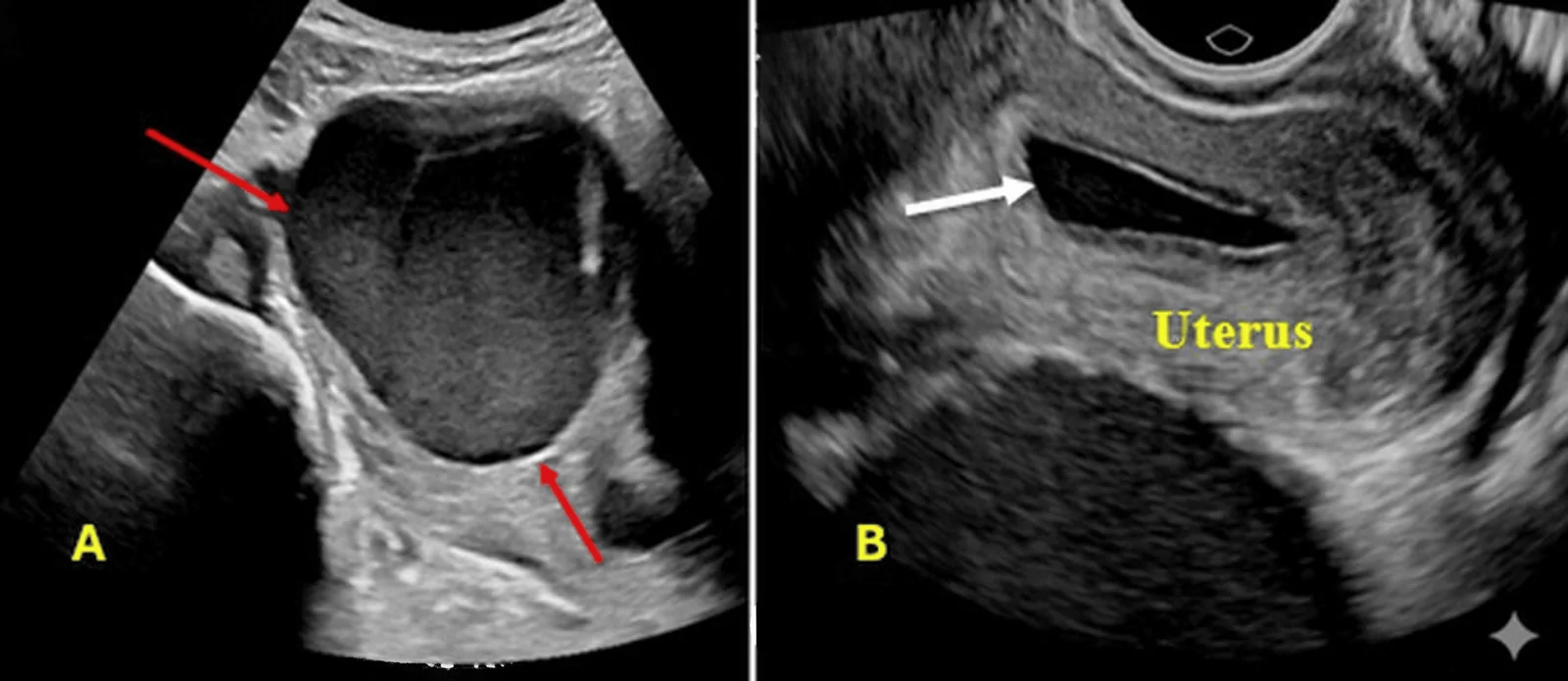
TVUS demonstrating hematosalpinx and endometrial fluid accumulation Red arrows indicate presumed severe hematosalpinx. Pyosalpinx is included in the differential diagnosis, as it may have a similar appearance. No evidence of ovarian torsion is identified. The white arrow shows a moderate increase in fluid within the endometrial canal. The cervix is not visualized. TVUS: transvaginal ultrasound

A repeat CT scan of the abdomen and pelvis (Figure 7) revealed severe bilateral hematosalpinx and marked accumulation of fluid within the endometrial canal, which was corroborated by another TVUS (Figure 8).

**Figure 9:**
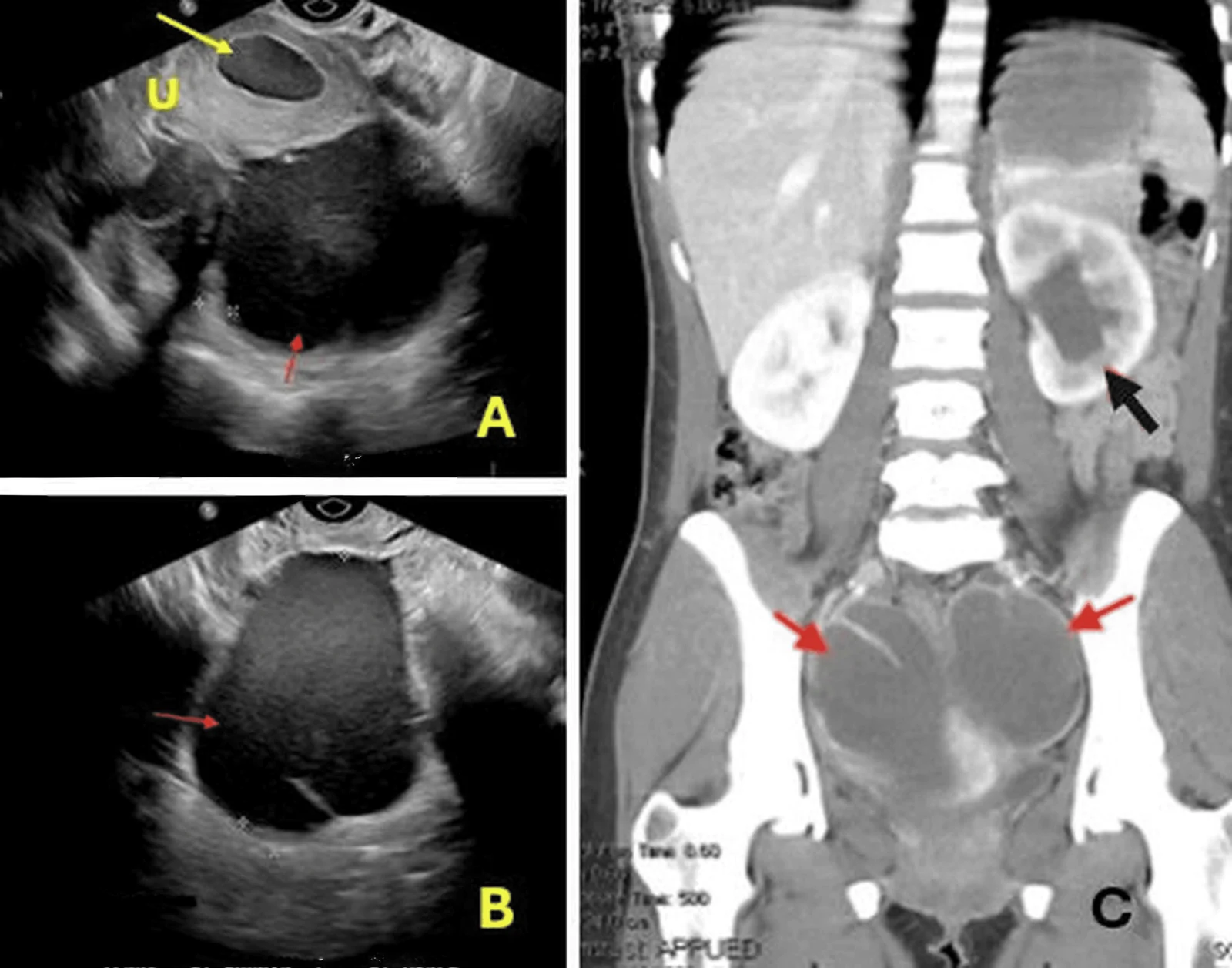
Follow-up pelvic imaging demonstrating progressive hematosalpinx and persistent left hydronephrosis "A" shows the left adnexa, and "B" shows the right adnexa. "U" indicates the uterus, and the yellow arrow indicates the fluid-filled endometrial canal. The red arrows indicate the pelvic masses. There is an interval increase in the volume of bilateral hematosalpinx, greater on the right than on the left, as well as increased hematometra. The left ovarian volume is 258.3 mL, compared with 224 mL previously, while the right ovarian volume is 694.7 mL, compared with 284 mL previously. "C" is a contrast-enhanced CT image of the abdomen and pelvis demonstrating persistent left hydronephrosis (black arrow) and hydroureter without an obstructing distal calculus. Bilateral complex, septated adnexal cystic lesions appear unchanged (red arrows), consistent with previously documented hydrosalpinx. TVUS: transvaginal ultrasound, CT: computed tomography

Crucially, the imaging revealed a new complication that had developed since the prior imaging approximately 18 months earlier. Secondary left ureteral obstruction with severe hydronephrosis and hydroureter, caused by extrinsic compression by the expanding pelvic masses, had developed. She was again discharged with instructions to follow up with the gynecology and urology services. Approximately five months after Day 0, she returned to the ED with persistent pain, where repeat imaging confirmed stable left hydronephrosis but an interval increase in the volume of bilateral hematosalpinx without evidence of obstructing nephrolithiasis (Figure 9).

During this final visit, the patient expressed profound confusion, stating that she believed her years of imaging had “never provided a definitive diagnosis.” She appeared to have limited appreciation of the importance of subspecialty follow-up. Despite an outpatient appointment being directly scheduled for her approximately six months after Day 0, the patient did not attend and was subsequently lost to follow-up within our healthcare network.

## Discussion

This case is important for two reasons. It illustrates a rare, devastating congenital anatomical anomaly and a profound, multisystem failure to maintain continuity of care for a vulnerable immigrant patient with LEP.

First, cervical agenesis with a functioning endometrium and primary amenorrhea is rare and can lead to significant morbidity from blocked menstrual outflow and retrograde menstruation [1,9,10]. The vast majority of cases are identified shortly after the onset of puberty, allowing timely, conservative surgical reconstruction of a functional outflow tract [11]. While surgical confirmation is definitive, a combination of precise physical exams and high-resolution pelvic MRIs can reliably establish the clinical diagnosis [12].

Our case is highly unusual given the patient's advanced age. She was 27 years old before her structural anomaly was first recognized on physical examination. In a comprehensive review by Fedele et al. of 257 patients with cervical agenesis across 138 publications, only two patients were older than 27 at the time of diagnosis [11].

Furthermore, the rapid onset of unilateral left-sided hydronephrosis underscores the progressive, dangerous nature of untreated retrograde menstruation over a decade. In this patient, the absence of urolithiasis confirms that the hydronephrosis resulted entirely from physical compression of the ureter by the expanding, blood-filled pelvic masses representing the fallopian tubes or surrounding endometriomas. This specific complication is exceedingly rare in the literature, as severe cyclic pain usually prompts definitive surgical or medical intervention long before the masses reach the size required to cause extrinsic ureteral compression. The only comparable case involved a 14-year-old patient whose delayed diagnosis required urinary diversion before a hysterectomy [13].

The second aspect of this case is the sequence of missed opportunities for early clinical intervention. The patient’s structural defect was first suggested by MRI approximately four months before Day 0, and a definitive speculum examination was not documented until approximately three months before Day 0. The initial encounters occurred entirely in emergency or primary care settings, where comprehensive pelvic examinations were repeatedly omitted, as documented in the reviewed records. Consequently, the care teams relied on passive outpatient referrals. Even after the diagnosis was clear, the patient did not attend a single subspecialty reproductive endocrinology consultation.

The literature consistently identifies LEP as an important factor associated with outpatient appointment nonattendance/nonadherence [14]. Over approximately 31 months, this patient attended 16 separate medical encounters across multiple independent health systems. Recent data confirm that such profound breakdowns in care continuity stem from systemic communication failures rather than intentional patient nonadherence [15]. Several systemic failures, including disconnected medical records across healthcare systems and a lack of clear assignment of post-discharge follow-up tasks, have been identified as potential contributors to the failure to complete recommended post-visit consultations [16]. More importantly, communication barriers, including handoff failures and unclear instructions to patients, especially for complex medical issues [17], have been identified. Our patient’s LEP likely compromised her understanding of her medical condition, discharge instructions, and appointment-scheduling protocols across different networks [18]. Translators were available, but Haitian women with LEP face unique systemic barriers, beginning with acute linguistic isolation and interpretation deficits [19].

In the absence of integrated cross-system tracking or navigation services, the patient was effectively left to navigate a siloed healthcare system, ultimately resulting in a complete failure to execute the recommended specialty care plan. Thus, her complex medical condition was never addressed beyond diagnosis, ultimately resulting in ureteral compression and the risk of renal damage.

Selected interventions that have been shown to improve follow-up attendance include using the "teach-back" technique, delivered via an interpreter, to confirm that the patient truly understands their diagnosis and follow-up needs [20]. Additionally, implementing specialized appointment reminder calls, automated messages, or navigators familiar with the system who use the patient's language can be beneficial.

This patient never received her subspecialty consult. In general, cervical agenesis with a functioning endometrium can be managed with conservative surgical treatment. The postoperative outcomes are generally favorable, with menstruation established in the majority of patients after restoration of continuity of the uterus, cervix, and vagina [3].

There are several limitations to this case report. This is a single-patient report, and the findings are descriptive and therefore cannot be generalized. Furthermore, there was no surgical confirmation. Finally, the report lacks the patient’s direct perspective, and follow-up was incomplete.

The absence of a dedicated patient navigator left her to manage a siloed medical system, ultimately exposing her to progressive renal risk and severe anatomical distortion. However, while sociolinguistic and systemic barriers may have disrupted continuity of care, successful health outcomes ultimately require a baseline level of patient adherence and individual responsibility to actively attend scheduled follow-up visits.

## Conclusions

Primary amenorrhea secondary to congenital cervical agenesis with a functioning uterus requires early detection and timely intervention to prevent the morbid sequelae of retrograde menstruation. This case demonstrates that congenital cervical agenesis can remain undiagnosed into adulthood, resulting in severe complications such as hematosalpinx and obstructive hydronephrosis. It underscores the importance of considering structural reproductive anomalies in patients presenting with primary amenorrhea, cyclic pelvic pain, and mass-effect symptoms. Additionally, this case highlights the profound impact of healthcare fragmentation and LEP on continuity of care, which contributed to delays in definitive treatment despite multiple emergency department and primary care presentations. Although conclusions drawn from a single case should be interpreted with caution, relying solely on passive referral workflows for patients with LEP may compromise timely access to specialty care. To reduce the risk of care failures and long-term organ damage, healthcare systems should consider implementing active, language-concordant patient navigation and interpreter-guided comprehension checks to facilitate appropriate follow-up and continuity of care.
